# The Hospitals and Surgeons of Paris, &c.

**Published:** 1844-10-01

**Authors:** 


### The Hospitals and Surgeons of Paris. An Historical and Statistical Account of the Civil Hospitals of Paris; with Miscellaneous Information and Biographical Notices of some of the most Eminent of the Living Parisian Surgeons. By *F. Campbell Stewart,* M.D. New York and Philadelphia.

This work contains a condensed account of the numerous and interesting hospitals and medical institutions of Paris, to which such general information has been added by the author, as to make the volume a useful manual or student's guide for such of his countrymen as might be disposed to visit that city for the purpose of completing their professional education. The entire work consists of two parts; the first part contains an account of the Hospitals and Medical Institutions of Paris; whilst, in the second part, will be found the lives of the most distinguished surgeons of that capital. Besides the information in regard to the several addresses and the hours of admission to the various places of medical resort, interspersed under different heads throughout the pages forming the first part, several Sections have been introduced, giving an ample account of the Schools of Medicine and Pharmacy, the Dissecting Rooms, Museums, &c. &c.

The administration of the Civil Hospitals of Paris, is conducted by 1st. A *General Council* consisting of seventeen members, of which the Prefect of Seine is President, and the Prefect of Police is a member *ex officio*. The other members are appointed by the King and serve for five years. This body holds a weekly meeting, and frames all regulations for the government of the public institutions. 2d. *An Administrative Committee*, consisting of five members of the General Council, who examine all accounts, &c. 3. *A Banker and a General Secretary*.

*Organization of the Hospitals*.—All the Civil Hospitals of Paris are divided into three classes:—1. General Hospitals; 2. Special Hospitals; and 3. *Hospices* or *Alms-houses*. Each hospital and most of the hospices, are provided, 1st, with a Director to superintend the general arrangements and internal police of the institution: 2nd, with *Surgeons* and *Physicians* proportioned to the number of beds in the hospital, about sixty patients being allowed to each. The surgeons and physicians are obliged to pay a daily morning visit to their patients; after which they prescribe in rotation for out-door patients. They are allowed one or more *internes* and *externes*, according to the extent of their service, one student of pharmacy, a nurse for each ward, and a Sister of Charity for each Service, male and female. Before being eligible to the office the surgeons must have attained the age of 30 years, and the physicians 35. The salaries vary from 600 to 1,800 francs, according to the length of their service. 3rd, with an *Apothecary*, who is chosen by *Concours*. 4th, with *Internes* or resident physicians—these too are chosen by *Concours*—they must have attained the age of 18 years, and have served one year as *Externe*; they must reside in the hospital, and their duty consists in visiting the patients in the morning with the surgeon or physician, and again by themselves in the evening, prescribing during his absence, and in cases of emergency, and assisting in all operations. They receive a salary of 400 francs and their board and lodging. 5th, with *Externes*, or " dressers," who are elected as the internes and serve four years. They do not reside in the hospital, nor do they receive any pay. 6th, with *Students of Pharmacy*. These are appointed after a *Concours*. The examination consists, 1st, in answering written questions on materia medica, pharmacy, and chemistry, for which *four hours'* time is allowed; 2, in answering oral questions, for each of which *ten minutes* is given; 3, in recognising such plants and articles of the Materia Medica as may be placed before them. Foreigners, as well as natives, are admitted to these *Concours*. 7th, with *Sisters of Charity*, who are members of some religious society. These reside in the hospital; they are divided into two classes, such as attend on the patients, and those who manage the internal arrangements of the hospital. The former have the entire charge of the wards during the absence of the surgeon or physician; they administer the medicines, and serve the patients with their diet. They receive only 200 francs per annum.

*Bureau Central.*—This is an office belonging to the General Administration. It is here that patients must apply for admission to hospital. To this office a certain number of medical men are attached, who are chosen by Concours, and from this body the hospital physicians and surgeons are selected.

Next follows a very full account of the Hospitals of Paris, of the number of beds, the physicians and surgeons annexed to them, &c.

In the account of the Physicians and Surgeons we very much desiderate in this work a piece of curious and interesting information which was furnished in a small French work brought out some years ago on a plan somewhat similar to the present by Dr. Ratier, entitled *Formulaire des Hôpitaux de Paris*, viz. an account of the peculiar opinions of the most eminent physicians and surgeons of Paris, either on the treatment of disease generally, or of some particular diseases to which they may have more especially devoted their attention. The author next gives an account of Medical Instruction in France; of the state of the Profession there; and a statement of the advantages of studying medicine in Paris. With respect to the state of the Medical Profession in France, he conceives it to be as elevated as in any other country of the world. One prominent cause of this he conceives to be the severe and rigid tests demanded of all applicants for the honour of admission into its ranks. A gradual increase has been made during the last ten years, in the quantum of knowledge required by the medical faculties. It is now made incumbent on all students to obtain a diploma of Bachelor of Sciences, before they can be permitted to undergo their fifth or final examination for a degree. This is somewhat similar to the requiring a degree of A.B. by the University of London of those persons who wish to obtain the degree of M.D. in that establishment. Instead of one general one, in France, students are subjected to five examinations, which, occurring at intervals of several months, and referring to such branches only as they shall have in the mean time studied, enables them to prepare themselves thoroughly to answer any question that may be put. The advantages attending the system of the *Concours* are greatly lauded; by it, they say, an equal and fair opportunity is afforded to all for making known their respective qualifications; and all who possess superior talent and acquirements, are sure of meeting encouragement and promotion. To the Concours Dupuytren owed, and Velpeau now owes, the elevation which they attained in their profession.

*Inscriptions and Qualifications required from Students.*—An inscription means the registering one's name in a register kept for the purpose. This has to be repeated every three months, and on each occasion the student will receive a card, certifying to the fact of his having inscribed. When a person who intends to study medicine, presents himself at the Bureau of the Faculty to take out his first inscription (which, as well as in the case of all subsequent ones, must be done in person), he is required to deposit with the Secretary the following documents:

1st. His certificate of birth.

2nd. His parent's or guardian's consent for him to study medicine, should he be a minor.

3rd. A certificate of his morality.

4th. His diploma of Bachelor of Letters.

A diploma of Bachelor of Sciences is required after the fourth inscription, and prior to the first examination.

The full term of study comprises four years, or sixteen quarterly terms.

*Examinations.*—Every pupil, before he can gain a diploma from the Paris Faculty of Medicine, must submit to five examinations, one of which takes place at the end of his first year of study, and the rest at stated intervals after. The first examination is on the subject of Chemistry, Physics, and Medical Natural History. The second, embraces Anatomy and Physiology. The third, Internal and External Pathology. The fourth, Hygiene, Legal Medicine, Pharmacy, Materia Medica, and Therapeutics. The fifth, and last, is a practical one, and is conducted at the hospital of the faculty (*Hôpital des Cliniques*); it consists in selecting two patients from the wards of the hospital and examining and prescribing for them in the presence of a committee of three professors. The first four examinations are conducted by two professors and one assistant professor, and three candidates are examined at the same time; for the fifth examination only two candidates at a time are admitted. Physicians and surgeons, graduates of Foreign schools, who may be desirous of obtaining a diploma from the Paris Faculty, must submit to all the examinations required from students; they must likewise exhibit their diplomas of Bachelor of Letters and Bachelor of Sciences. If they can show, by proper certificates, that they have studied in a Foreign University during two years longer than is required in the Paris Faculty, they are entitled to an immediate examination, on the payment of all the charges exacted for the five separately. If they cannot produce proof of having been six years engaged in study, then such time as they have studied will be allowed them, in the proportion of two-thirds.

The King has the privilege of granting unconditional licences to foreigners to practise in France. The examinations are conducted in French, and by the professors themselves, or by the assistant-professors (agrégés). A student is always notified, four days before-hand, of the time appointed for an examination; and, if he do not present himself at the hour indicated, he is not allowed to apply for another examination until after the expiration of three months, which are thus lost to him.

*Theses.*—The Theses of all candidates for medical degrees are to consist of written answers to four questions, which are to be drawn for by lot. The questions embrace the subjects of the physical sciences, anatomy and physiology, surgery and medicine. The thesis is to be deposited in the hands of the Dean when the student offers himself for his final examination, and it is referred to some member of the faculty to examine, prior to its being supported in public by the author. All these must be printed at the expense of the student. The whole expences incident to obtaining a medical degree from the Paris Faculty amount to eleven hundred francs.

The remaining portion of the First Part of this work contains succinct accounts of the various Medical Societies in Paris, of the Medical Journals published there, with the names of their editors, &c. To this is annexed an account of the expences and mode of living in Paris. The information here given is of the greatest possible use to the stranger who goes to Paris for the first time. The principal hotels, with their prices, the lodging-houses, boarding-houses, &c. are here described with such precision and clearness, that no one can go astray in this particular who has this volume to consult. The First Part closes with a Bibliography, or an account of the principal medical and surgical works, with the names of the authors.

The Second Part is devoted to Biographical Notices of some of the most eminent Parisian Surgeons. The biography of living men is a very ticklish sort of thing. Whilst distinguished merit is still present amongst us, it is exposed to all the disadvantages of misrepresentation, misrepresentation, however, arising less frequently from the partiality of friendship, than from the malignancy of envy:

*Urit enim fulgore suo qui prægravat artes*

*Infra se positas : extinctus amabitur idem.*

In conclusion we have to say that the medical student who intends going to Paris, in order to complete his medical education, will find his account in providing himself with this book.

---

